# Identity, stigma, and HIV risk among transgender women: a qualitative study in Jiangsu Province, China

**DOI:** 10.1186/s40249-019-0606-9

**Published:** 2019-12-01

**Authors:** Zi-Han Yan, Jessica Lin, Wen-Jing Xiao, Keh-Ming Lin, Willi McFarland, Hong-Jing Yan, Erin Wilson

**Affiliations:** 1 University of California, Berkeley, USA; 2 San Francisco Department of Public Health, 25 Van Ness Avenue, Suite 710, San Francisco, CA 94102-6033 USA; 3 Jiangsu Provincial Center for Disease Control and Prevention, 172 Jiangsu Road, Gulou District, Nanjing, 210009 Jiangsu China; 4 Nanjing Medical University, Nanjing, China; 5 University of California, Los Angeles, USA; 6 University of California, San Francisco, USA

**Keywords:** Transgender women, China, Sexual and gender minorities, Gender identity, Stigma, Discrimination, HIV

## Abstract

**Background:**

Transgender women have multiple disparities globally, including social rejection and stigma, HIV infection and untreated mental health problems. However, few data on transgender women are available in China. Therefore, this study aimed to explore transgender women’s experiences on gender identity, disclosure, discrimination, transgender-specific medical care, and perceptions of HIV and sexually transmitted infections (STI) risk in China.

**Methods:**

A qualitative study was conducted in Nanjing and Suzhou city, China in 2018. Key informant interviews (*n* = 14) and focus group discussions (*n* = 2) with diverse transgender women were implemented. Text was transcribed and translated, and *Dedoose*™ software was used for coding, analysis and interpretation by the research team.

**Results:**

Chinese transgender women share experiences with transgender women worldwide, including a long and challenging identity search, stigma and discrimination, poor access to trans-specific services and unmet needs for mental health care. Features unique to them include terms used for self-identification, culturally-shaped expectations for reproduction, and ideals of placing the familial and societal welfare over personal fulfillment. Social networks of this population appear sparse, scattered, and underground. Familial rejection was experienced by nearly all respondents. Perceptions of HIV and STI risk and history of HIV testing were notably low.

**Conclusions:**

Transgender women in China face high social rejection and discrimination along with unmet need for various types of healthcare. Scaling up transgender-specific services including gender-affirming medical care, mental health care and HIV/STI prevention are warranted to address the social, medical and mental health of transgender women in China.

## Multilingual abstracts

Please see Additional file 1 for translations of the abstract into the five official working languages of the United Nations.

## Background

Transgender women are person assigned male sex at birth who self-identify as women or another feminine gender [1–3]. Although transgender women have been documented in many cultures for thousands of years, the population size and distribution of transgender women worldwide is unclear due to stigma, marginalized status, and limited attention in research [4]. Estimates of the proportion of transgender people within populations across the world ranged between 0.1 and 1.1% of reproductive age adults [5]. Example estimates include 700 000 transgender women living in the United States and 314 808 in Thailand [6, 7].

Previous studies have documented that transgender women carry a disproportionate burden of HIV infection [1, 8–10]. Transgender women experience high levels of multiple factors related to HIV risk, including substance use, sexual abuse, condomless sex, engagement in sex work, and lack of access to HIV testing, care, and prevention [2, 5, 11]. Transgender women may in fact be the population most severely affected by HIV worldwide. A meta-analysis from 15 countries estimates a pooled HIV prevalence of 19.1% among transgender women (95% *CI*: 17.4–20.7) and a 49-fold odds of infection compared to the general population [8]. In China, where few data specifically on transgender women are available, an online survey targeted to men who have sex with men (MSM) that also included transgender women showed a higher level of self-reported HIV infection among transgender women compared to MSM (11.7% vs 5.7%) [11].

Social rejection and stigma are very common experiences for transgender women. Numerous studies have documented that transgender women experience intimate partner violence, physical abuse, rejection by parents and family, harassment or violence from police officers, and negative attitudes from service providers due to their gender identity or expression [12–14]. Transgender-specific care and support are also insufficient, contributing to significant health disparities [15, 16], particularly for mental health [13, 17]. A United States (US) sample of 1093 transgender persons found a high prevalence of clinical depression (44.1%), anxiety (33.2%), and somatization (27.5%) [18]. An online survey in China also found high rates of depressive tendencies (29.4%), severe anxiety (21.1%), self-mutilation (21.5%), and attempted suicide (12.7%) [19].

The small body of research that has been conducted to date with Chinese transgender women indicates multiple disparities, including HIV infection and untreated mental health conditions [11, 19]. There is a paucity of basic information on gender identity, disclosure, discrimination, and barriers to transgender-specific medical interventions and health care. In order to start to bridge these important gaps in the literature, we conducted a qualitative study in two cities in Jiangsu Province, China. With this study, we strive to provide a snapshot of the lives of contemporary Chinese transgender women through the eyes of diverse key informants, explore the terminology of gender identity, describe their social networks, gauge their access to transgender-specific care and support, and better understand their sexual behavior and perceptions of HIV risk.

## Methods

### Study design and population

Between January and March 2018, a qualitative assessment among transgender women was conducted in Nanjing and Suzhou cities in Jiangsu Province, China. Eight transgender women key informants in Nanjing and six in Suzhou were interviewed. Participants were referred by leaders working in local grass-root non-governmental organizations (NGOs) in the transgender women community. Leaders described the study to potential key informant participants and asked consent for the study team to call them. All potential participants agreed to share their information and participate. Leaders gave mobile phone information to the study team who then called each potential formative participant and scheduled a time to meet for participation in the key informant interview. The key informant interviews (KII) informed questions for two subsequent focus group discussions (FGD) involving the same participants in each city. Purposive sampling was used to recruit a diverse group of transgender women by age, education, and occupation. Interviews with formative participants were conducted in local NGO offices and club venues transgender women frequented and felt most comfortable participating. All interviews were audio recorded. Field notes were also taken to ensure all questions were asked as part of the in depth interview. To be eligible, participants had to be 18 years old or older, have been assigned male sex at birth, and identify as a woman or along the feminine spectrum. Written informed consent was obtained. An incentive of 200 RMB (~ 30 USD) for KII and FGD participation was given to compensate for time and transportation.

### Procedures, measures and quality control

A semi-structured interview guide was developed by the investigators from the Jiangsu Provincial Center for Diseases Control and Prevention and the San Francisco Department of Public Health in California, USA. Transcription, translation, coding, analysis, and interpretation were done by the team in China and the US. Interviews were audio taped and transcribed verbatim in Chinese. The principal investigator of the Chinese site and two bilingual research assistants performed English translations, checking for discrepancies and reconciling by consensus. A general codebook was built based on considering the above-listed domains for KII and FGD questions and the content of the transcripts. Two coders did a pilot coding on one transcript to test, modify, and finalize the codebook in consultation with all investigators. They then further used the key word search to find core information related to the major themes and coded the all transcripts independently.

The KII guide was structured to cover several domains, including: (1) social networks and transgender women communities; (2) violence and discrimination towards transgender women; (3) gender identity, hormone use, and gender-related surgeries; and (4) mental, physical, and sexual health. The FGD guide covered: (1) terms used to identify transgender women in the community; (2) mapping places where transgender women socialize; (3) laws and services specific for transgender women; (4) health and medical transitioning; and (5) perceptions of HIV risk. All interviews were conducted by trained program investigators in a private room to maintain participant privacy. Investigators reviewed transcript coding for consistency with the codebook and code definitions to ensure quality of the data analysis.

### Qualitative analysis

*Dedoose*™ qualitative app version 8.0.35 software (SocioCultural Research Consultants, LLC, Los Angeles, USA) was used for data analysis. Analysis and interpretation entailed identifying common patterns and frequently expressed themes within and across the pre-determined domains. The lead author took the first pass at synthesizing and consolidating interpretations. Input was iteratively obtained from the other investigators in China and the US. Quotations are assigned to unique individuals by fictitious initials and a real age range.

The study was approved by the internal review boards of the University of California San Francisco and the Jiangsu Provincial Center for Diseases Control and Prevention.

## Results

### Demographic characteristics

Fourteen transgender women participated in the KII and FGD at the Nanjing and Suzhou research sites (Table 1). Participants were 20 to 55 years of age, 42.9% had their *Hukou* (official registered domicile) outside of Jiangsu, indicating internal migration, but all currently resided in the province. All participants were single; 42.9% had a bachelor degree; 49.2% had some college or technical degree; 14.3% had high school education or lower. Most (92.9%) were employed, had regular income, and were financially self-supportive, with only one (7.1%) still in school and receiving assistance from a shelter. Of those employed, approximately half (46.2%) worked in nightlife-related jobs, principally as stage performers. All participants identified as transgender women, but only 35.7% of those lived openly as such.

**Table 1:** Demographic characteristics of transgender women participants in Nanjing and Suzhou City, Jiangsu Province, China (*n* = 14)

	*n* (%)
Age: Median/range (SD)	25.50/20–55 (9.271)
Hukou	
Nanjing City	2 (14.3%)
Suzhou City	5 (35.7%)
Jiangsu Province	1 (7.1%)
Other	6 (42.9%)
Marital status	
Single	14 (100.0%)
Married/divorced	0 (0.0%)
Education	
High school or lower	2 (14.3%)
Some college or technical school	6 (42.9%)
Bachelor/College	6 (42.9%)
Occupation	
Full time job	4 (28.6%)
Self employed	3 (21.4%)
Nightlife-related job	6 (42.9%)
Student	1 (7.1%)
Assignment gender at birth	
Male	14 (100.0%)
Female	0 (0.0%)
Internal sense of gender identity	
Male	0 (0.0%)
Female	14 (100%)
Lived as the gender identified	
Sometimes	9 (64.3%)
Always	5 (35.7%)

### Terms of transgender women

Participants described transgender identity using multiple key terms imported and translated from the West and Southeast Asia and reconceptualized into Chinese terminology and sensibilities (Table 2). These terms included *kua xing bie zhe*, *bian xin gren* (the Latin letters “TS” are equivalently spoken and written), and *yi zhuang zhe* (the Latin letters “CD” equivalently spoken and written). The majority of participants accepted being called *kua xing bie zhe* and recognized it as a neutral term. However, many reported negative feelings towards the terms *bian xing ren* and TS, *yi zhuang zhe* and CD, as well as the term *ren yao*. In one focus group, several transgender women discussed their resistance to using the term TS or CD because it had become associated with those engaged in commercial sex work.

**Table 2:** Glossary of terms referring to transgender women in Jiangsu Province, China

Pinyin	Literal	Meaning	Acceptance
*kua xing bie zhe*	Transgender	People whose gender identity differ from the biological sex assigned to them at birth	Neutral
*bian xing ren*	Transsexual	Transgender women who engage in sex work, used in the title of dating apps	Negative
TS	Transsexual	Transgender women who engage in sex work, code used in the title of dating apps	Negative
*yi zhuang zhe*	Cross-dresser	Transgender women who engage in sex work, used in the title of dating apps	Negative
CD	Cross-dresser	Transgender women who engage in sex work, code used in the title of dating apps	Negative
*ren yao*	She-male/ lady boy	Humiliating term, refers to imps or goblins in ancient literature	Negative
MTF	Male to female	Abbreviations of male to female	Neutral
*hong yi ren*	Red artists	Beautiful stage performers	Positive
*yao niang*	Drug girl	Transgender women who need/use hormone therapy to maintain feminine traits	Positive
*jie mei*	Sister	A friendly reference to other transgender women, mainly used within the group	Positive

To some degree, most key informants agreed with the terms used to define and label the subgroups they identified with. About one fifth (21.4%) had never engaged in sex work and preferred to call themselves *kua xing bie zhe* and MTF. The 42.8% who had worked as CD performers preferred the term *hong yi ren*. Younger transgender women who took hormone therapy or had gender-affirming surgeries preferred the term *yao niang*. There appeared to be a consensus that the term *jie mei* is the best way for them to address each other.

Of note, more than one respondent complained about the potential of these terms artificially separating people into different camps, creating divisions and fostering a sense of alienation inside the transgender community, as indicated by the quotation below.

*Trans includes many people. If you are too particular on subdividing, you may alienate people emotionally and psychologically. (SV, 25–35 years old)*

### Self-realization of gender identity

Participants realized their gender identity at an early age. Most reported that they detected an inconsistency between their assigned sex and gender identity in early childhood, and began to question their gender identity while in kindergarten or elementary school. One story of feelings of inconsistency and a moment of self-realization is particularly illustrative:

*I wrote entries in my diary at that time. One day I drew an image. The image showed a small person figure [you usually see] outside men’s rooms, a male sign. At the same time, I drew a female's sign at the place of the heart. Then I realized that I was this girl in my heart. (WX, 18–24 years old)*

Many participants reported being enlightened by information available from news, television, and the internet. Online searches using keywords such as *kua xing bie zhe*, *yi zhuang zhe*, and *yao niang,* and entry into online socialization forums including *Zhihu*, Wechat, and *Tieba*, gave many participants their first access to information about transgender communities. One participant mentioned watching Li Yu-Gang (a Chinese male opera performer famous for playing female roles) on television:

*I never thought about and had no idea of the potential feminine inclination inside of me, not until I saw people like Li Yugang. A man could be so beautiful after makeup. Many people also said, “Wow, you look like a girl”. Then, I behaved more and more like a girl, and it slowly took me to the road of transgender women. (CC, 18–24 years old)*

Some transgender women benefitted from the guidance of “teachers” (senior, more experienced transgender women). One participant described behaving as a teacher mentoring other transgender women through the discovery of their gender identity:

*My friend who did not join the transgender community knew nothing about it before. She bought contraceptives and started using them. I caught her doing this and I told her this was not good. I sent her information on transgender identity and the correct way for doing it. She probably is doing well with her new life. (SV, 25–35 years old)*

### Transgender-specific medical care

Transgender-specific medical care desired by participants was gender-affirming surgeries, identification (ID) changes that are legally permitted only after vaginoplasty is complete, hormone access, and gender-related knowledge of health care providers. Only one of the fourteen participants had vaginoplasty. Many said that they were unable to access hormones from a medical provider and lacked appropriate guidance on how to use the medications. As a result, transgender women were buying and administering their own hormone therapy regimens with no medical guidance, which further worsened their health. A participant shared her experiences of taking hormones without medical consultation:

*At the age of 18 or 19, I took hormones for two months. However, it disturbed my endocrine system and caused emotional problems. I became very bad tempered, and suffered from hair loss. At that time, there were 100 tablets in one bottle and I would take all the 100 tablets [sic]. I didn’t consult doctors, I rushed for quick results, like taking diet pills. (PM, 25–35 years old)*

### Expression and disclosure of gender identity

Participants indicated different degrees of gender identity expression in public and private settings. Most reported the desire for permanent social and medical transition, yet they tended to wear gender-neutral clothing, maintain gender-neutral appearances, and speak with a masculine pitch in public and with parents and friends. They reported behaving and dressing in a more feminine manner in private settings, with their partners and with other transgender people. Participants’ choice of public restroom was dependent and aligned with their gender presentation at the moment.

Most participants wanted to disclose their gender identity to their parents (93%) and close friends (74%), but not to other relatives, municipal authorities, or medical providers. Disclosure was often done online, but in an anonymous way by using an online profile name different from their real name. Some transgender women described adding the terms described above to their online profile names, such as *yao niang* and MTF. They did this to signal and meet new people in the community while concealing their identity for privacy. Other participants reported barriers to disclosing their gender identity such as lack of family and social support, legal challenges to changing national ID documents, and economic risks. As an example, a transgender business owner described the economic costs she could face for disclosing her gender identity:

*Although I myself can disclose my identity, but at the workplace, I have my employees, I have my business partners. If my gender identity is exposed, many major partners of mine are likely to be affected (RM, 25–35 years old)*

### Family rejection due to gender identity

All participants stated that they desired familial support. Though family attitudes varied, most reported rejection, physical violence, verbal abuse, and suppression and restriction of their gender-related expression by family members. Parents’ desire for their child to get married was a key reason for harsh reactions to their child’s gender identity. Conflicts with parents became extreme for those who were an only child and were blamed for shirking the responsibility for continuing the bloodline. One participant shared:

*Many parents still have great hopes if it is only about sexual orientation, because they think it could still be treated medically, and doesn’t involve operations on bodies. But transgender women basically need surgery on the bodies. Then for parents, either out of concerns about their children’s health, or because of the influences of traditional cultural values, it is hard for them to understand, to accept. (JK, >35 years old)*

Participants who disclosed their gender identity to their parents reported being hurt by the lack of understanding and intolerance and their parents’ trivializing their struggles with identity as naughty behavior. Some even experienced violence from family.

*My dad, my dad, and he will keep asking me, asking if I am a man or a woman. When I refused to answer, he slapped me. Every time I refused to answer, he slapped me, throwing me down directly on the ground and beat me. (QQ, 18–24 years old)*

### Harassment, violence, and discrimination

Discriminatory experiences related to transgender identity were prevalent. Most experienced difficulties accessing hotel accommodations and opening bank accounts due to ID issues, as their outer appearance did not match their male ID. They were often subject to hostile attitudes towards their appearance and clothing. In order to avoid discrimination from society, concealment became an often-used strategy. As one participant described:

*When you get used to people’s staring and gossiping, you learn how to avoid being hurt. So, as I said, I go out without makeup during the day, when my voice is good, I use a girl’s voice to talk, when the voice is bad, I use a boy’s voice to talk. (PM, 25–35 years old)*

Discrimination at work was also common. Several participants reported suffering from discrimination aimed at women in general, including disrespect and harassment, as well as disadvantages in gaining employment as compared to males. Some reported experiences of being robbed or sexually harassed. Some had been forced into uncomfortable or discriminatory situations by police officers, and did not trust the police.

### Social networks

The social networks of transgender women varied in composition and size, depending on occupation and social skills. Most participants indicated that they were networked with transgender, gay, and lesbian social circles. On average, participants’ social circles included roughly ten other transgender women in the community. Transgender women who performed as CD had comparatively larger social circles, knowing an average of 30 other transgender women through work. Several websites and apps such as *Zhihu*, *Momo,* transgender-specific QQ and Wechat provided means for socializing virtually, including sex work-oriented online groups. The few off-line gathering places available to transgender women were nightclubs and gay and lesbian bars.

The community ties among transgender women were loose. High mobility of transgender women was also discussed by some participants. When explicitly asked if there was a transgender community, responses were often equivocal. For example, one stated:

*A2: I don't know. Because for transgender women, the first thing is that there are not many of them, and the other is they have higher mobility and tend to hide themselves. (FGD, RX, 25–35 years old)”*

They reported that senior transgender women tended to drift away from the community. After completing social and medical transition, some established families and their lives became more stable, with less need for support from the community.

### Mental health concerns

Many participants self-reported psychological challenges and mental illness, including gender dysphoria, depressive thoughts, distress, anxiety, suicidal attempts, and self-mutilation. Most depressive, distressful, and anxious thoughts were associated with inability to obtain medical transition services and the frustration over not being able to achieve their transition-related goals. These symptoms were exacerbated when in contact with transgender women who had accessed gender-affirming surgeries or hormones and who had boyfriends or girlfriends. Also affecting their mental health were family abuse, social discrimination, economic struggles, and domestic violence. Several study participants reported histories of suicidality, alcohol addiction and dependence, and self-mutilation resulting from extremely distressing thoughts and emotional burdens. One participant described her persistent suicidal thoughts as a wish that suicide might give her another chance to live a better life:

*How good it would be to die and restart! There are a lot of frustrations: when you suffer from violence at home, when you are being discriminated against, or when you see the “sisters” in your circle, one after another, getting to go for surgery and you still could not have the surgery yet. (QQ, 18–24 years old)*

### HIV and sexually transmitted infections risk perceptions

Overall, participants were not very concerned about risk for HIV and other sexually transmitted infections (STI). Very few participants felt transgender women regularly tested for HIV and other STI, reporting lack of adequate understanding in the community, difficulty finding convenient testing sites, and fear of discrimination and stigma at testing sites. One participant described:

*Many people do not know HIV. They do not know how to protect themselves, how to get treatment after infection with HIV, and how to cope with the stress, and the most important thing is to know what happens after getting infected with HIV, they think they are dying. (WG, 18–25 years old)*

None of the participants reported being HIV positive, and only one self-reported having previously tested positive for an STI. Anal intercourse, oral intercourse, and vaginal sex with women were the sexual behaviors reported by most participants; however, they expressed a relatively low attention to HIV in the community.

## Discussion

Chinese transgender women in our province and study share with their counterparts worldwide the long and challenging identity search process , stigma and discrimination, difficulty accessing trans-specific healthcare, stresses and mental health consequences, and scarcity of expertise and resources to address their needs. Nuanced differences between Chinese transgender women in Jiangsu and other transgender women are terms used for self-identification, culturally-shaped expectations for reproduction, and the ideals of placing the familial and societal welfare over personal fulfillment.With respect to identity, these interviews uncovered a diverse set of terms that reveal how transgender women define themselves and regard others in their community. While some terms approximate direct translations of recently imported words, others emerged locally, reflect Chinese cultural traditions, and take divergent connotations. The imported terms, *kua xing bie zhe* and MTF are regarded as neutral. Labels used only in Chinese communities include *yao niang* and *hong yi ren* are regarded as positive. *Bian xing ren* and *yi zhuang zhe* have negative connotations. *Yi zhuang zhe* in particular is associated with sex work. The term *ren yao* has appeared in Chinese texts for more than a millennium. As its literal translation implies, *ren yao* is strongly derogatory. In the context of its use, it is more figuratively the Chinese equivalent of “lady-boy” or “she-male”. Although familiar with these terms, respondents voiced strong objection to many as divisive, alienating, and traumatizing to members of the community.

Compared to gay communities in many parts of China, where there are multiple online sites and in-person venues for socializing [20], the social networks of transgender women appear sparse, scattered, and further underground. Chinese transgender women rely mostly on the internet to socialize, meet friends, and seek sexual partners. Some report venturing into gay bars without revealing their gender identity. Moreover, transgender women who have completed their intended gender transition goals may distance themselves from the community and one another. Transgender women’s social circles are subsequently small. Most count knowing around ten other transgender women, including those with whom they only interact online. Social isolation makes it difficult to reach transgender women to provide health education, HIV testing, and prevention interventions.

Social isolation is reported to begin at a very early age and continue to adulthood. Recognition of gender incongruity occurred for most informants around kindergarten or primary school. Yet, many lacked the framework and language to clearly identify themselves as transgender women until in their twenties. Lacking easily available guides, they searched for information haphazardly, culling from the news, television, and internet. Such sources may not be scientifically sound, reliable, or supportive. This is regrettable, since delays in transition are correlated with negative health outcomes in transgender women [21]. The outward presentation of their gender identity is also quite variable, even within a typical day. For long periods of their lives, many maintain a male appearance in public and show their female persona only in private settings. Although most informants had disclosed their gender identity to their parents and friends, they typically did not feel accepted by them and felt pressure to present as male even to close family.

Parental rejection was a common and highly emotional theme for respondents. Even though many no longer lived with their parents, participants continued to have frequent, intense, and stressful interactions with them. As most parents lived outside of metropolitan areas and grew up with much more limited access to the outside world compared to their children, it may be expected that they experience difficulty understanding and accepting their children’s aspirations and struggles. Moreover, they were more likely to embrace traditional Confucian thoughts on the importance of having descendants [22]. The One Child Policy, implemented in 1979 and enforced until 2013, likely made these conflicts worse [23]. Although dealing with social pressure is a challenge for transgender women everywhere, such burdens may be particularly heavy for those living in more collectively oriented societies, such as China.

Across interviews and focus groups, the most frequently discussed topic was poor access to trans-specific medical care. Nearly all participants desired vaginoplasty, but only one had completed the surgery, which was done in Thailand. Low access to gender-affirming surgery among Chinese transgender women is consistent with Jiang’s estimation that fewer than 800 transgender patients have been treated in the past 30 years in China [24, 25]. As with transgender women in other countries, obstacles include the high cost of surgery, scarcity of providers with expertise in transgender medicine, and limited availability of hormone therapy, which was only available through a few high-tier hospitals [18, 26]. A particular barrier to gender reassignment surgery in China is the requirement of familial approval [25]. The requirement is interpreted that parents or other kin of the family (parent death) must approve at any age - an interpretation that is held by hospitals, physicians, authorities, and transgender women themselves. The requirement further compounds the familial stresses mentioned above.

Similar to previous studies [13, 19], discriminatory experiences are highly prevalent among participants. They suffer from disrespect, robbery, sexual harassment, and job discrimination due to their gender identity. Difficulties in finding employment and generating income are major contributing factors for transgender women’s high rate of involvement in commercial sex work [26]. A survey of transgender persons in the United States in 2015 found that 19% of respondents were engaged in some type of sex work for money, food, or a place to sleep [13]. Globally, transgender women sex workers experience significantly higher risks for HIV infection in comparison to other groups [27].

Possibly resulting in part from discrimination, financial strain, social rejection, or medical challenges, our respondents described very high risks of developing psychiatric disorders in their communities. Poignantly, they indicated high levels of depression and attempted and completed suicides. Our study suggests that the depressive thoughts and suicidal behavior may be associated with the frustration and pain of being unable to access appropriate medical transition services, often due to family pressure, lack of available domestic services, and the high cost of surgery [24]. Further research is needed to systematically search for factors that may be predictive of risks for psychiatric and mental disorders, and to find ways to address these problems.

Congruent with other studies [11, 28], many of our respondents were unsure of their HIV status and expressed that preventive behaviors were a low priority in the community. Correspondingly, HIV testing was low and inconsistent. Although measuring HIV prevalence was not the focus of this qualitative study, our data indicate a discrepancy between perception of risk and behaviors. This is particularly worrisome since anal intercourse without condoms was common. Respondents also indicated sexual partners include gay men. Chinese transgender in our study appear less engaged in HIV prevention than would be indicated by international data showing extraordinarily high HIV prevalence among transgender women globally [29].

This study has several limitations. First, by design this was a qualitative study with a small, purposively selected sample of transgender women. Although participants were diverse in terms of age, education, occupation, and residence in two cities, the findings may not be generalizable across the vast expanse of China. Second, while participants shared personal information enthusiastically throughout in-depth interviews and in groups, they may still have been hesitantly respond candidly to some of the more sensitive questions, such as HIV status and involvement in commercial sex work. Third, as with many aspects of contemporary Chinese society, the situation for transgender women may be in a period of rapid change. Our data may therefore represent a transient moment. Finally, the structure of our guides may have missed other salient aspects of the lives of Chinese transgender women. Despite these limitations, this study provides unique and novel insights into the ways in which health and identity intersect creating high vulnerability to HIV for transgender women in China. Contributions from this analysis are the ways in which transgender women identify to help find them and engage them in research and data to drive intervention development that addresses the high stigma and discrimination they face.

## Conclusions

In summary, our study demonstrates that transgender women in China face high social rejection and discrimination along with unmet need for various types of healthcare. Since transgender awareness in the modern sense in China is still in early development, there may be opportunities for researchers, services providers, and advocates to inform and shape clinical practices, infectious disease control services and policies that can optimally benefit the transgender population. Future studies can corroborate or refute our findings, expand upon areas missed, and implement larger studies of different designs, including surveys for HIV prevalence and novel interventions to address the social, medical, and mental health needs of transgender women in China.

## Supplementary information

**Additional file 1.** Multilingual abstracts in the five official working languages of the United Nations.

## Data Availability

The datasets used and/or analyzed during the current study are available from the corresponding author on reasonable request.
