# Supplementary material for: Identity, stigma, and HIV risk among transgender women: a qualitative study in Jiangsu Province, China
Source: Infect Dis Poverty. 2019 Dec 1;8:94. doi: 10.1186/s40249-019-0606-9 (PMC6885322; doi:10.1186/s40249-019-0606-9)
Supplement: Supplementary file 1 — Additional file 1. Multilingual abstracts in the five official working languages of the United Nations. [file 40249_2019_606_MOESM1_ESM.pdf]

Translation of the abstract into the five official working languages of the United Nations

الهوية، وصمة العار ومخاطر فيروس نقص المناعة البشرية لدى النساء المتحوّلات جنسياً: دراسية نوعية في مقاطعة جيانغسو، الصين.

زيهان يان، جيسيكا لين، وينجنغ شياو، كي-مينغ لين، ويلي ماكفارلاند، هونجينغ يان، إرين ويلسون

نبذة

المعلومات الأساسية:تواجه النساء المتحوّلات جنسياً العديد من الفوارق عالمياً، بما في ذلك الرفض الاجتماعي ووصمة العار، عدوى فيروس نقص المناعة البشرية، ومشاكل الصحة العقلية الغير معالجة. مع ذلك، لا يوجد إلّا عدد قليل من البيانات المتاحة حول النساء المتحوّلات جنسياً في الصين. لهذا السبب، تهدف هذه الدراسة إلى استكشاف تجارب النساء المتحوّلات جنسياً فيما يخص الهوية الجنسية، الإفصاح، الاضطهاد، الرعاية الصحية الخاصة بالتحول الجنسي، والتصورات المتعلقة بمخاطر عدوى فيروس نقص المناعة البشرية والأمراض المعدية المنقولة جنسياً في الصين.

المنهجيات: تم إجراء دراسة نوعية في نانجينغ وفي مدينة سوجو في الصين في عام 2018. تم تنفيذ المقابلات مع المستجيبين الرئيسيين (n = 14) و مناقشات مجموعات التركيز (n = 2) مع مجموعة متنوعة من النساء المتحوّلات جنسياً. تم استنساخ مضمون النصوص وترجمتها، واستخدام برنامجي Dedoose™ للبرمجة والتحليل والتفسير من قبل فريق البحث.

النتائج: تتشارك النساء المتحوّلات جنسياً في الصين ذات التجارب مع النساء المتحوّلات جنسياً في انحاء العالم، وتتضمن تلك التجارب المشتركة البحث الطويل والصعب عن الهوية، وصمة العار والاضطهاد، إمكانية الوصول السيئة للخدمات الخاصة بالمتحولين جنسياً وعدم تلبية حاجات الرعاية الصحية العقلية. أما المميزات التي تميزهم، فتتضمن المصطلحات المستخدمة للتدليل على الذات، التوقعات المشكلة ثقافياً حول الإنجاب، والمثل العليا التي تفضّل المصلحة العائلية والاجتماعية على الرضى الفردي. تظهر الشبكات الاجتماعية الخاصة بتلك الشريحة السكانية ضئيلة ومشتتة وسريّة. عانى تقريباً كافة المستجيبين في الدراسة من الرفض من قبل العائلة. تبيّن أن كلاً من التصورات المتعلقة بمخاطر فيروس نقص المناعة البشرية والأمراض المنقولة جنسياً والإقبال على فحوصات الكشف عن فيروس نقص المناعة البشرية كانت منخفضة بشكل ملحوظ.

الاستنتاجات: تواجه النساء المتحوّلات جنسياً في الصين درجة عالية من الرفض الاجتماعي والاضطهاد بالإضافة إلى الاحتياجات غير المستوفاة للعديد من أنواع الرعاية الصحية. زيادة الخدمات الخاصة بالمتحولين جنسياً بما في ذلك الرعاية الصحية المتعلقة بعمليات التحول الجنسي، والصحة العقلية، والوقاية من فيروس نقص المناعة البشرية والأمراض المنقولة جنسياً، مبررا لمعالجة الصحة الاجتماعية ، الطبية والعقلية للنساء المتحوّلات جنسياً في الصين.

Translated from English version into Arabic by Suzanne Zaza, revised by Mohamed Aberzak, through

TRANSLATORS
WITHOUT BORDERS

中国江苏省跨性别女性人群性别认同、歧视性及 HIV 感染风险定性研究

闫子寒，Jessica Lin，肖文静，林克明，Willi McFarland，闫红静，Erin Wilson

摘要

引言：跨性别女性在全球范围内存在多种差异，包括社会排斥和歧视性，艾滋病感染以及未经治疗的心理健康问题等。我国在跨性别女性人群中的研究报道甚少。本研究旨在探讨中国跨性别女性人群在性别认同、身份披露、社会歧视及寻求跨性别医疗服务过程中的经历以及该人群对艾滋病和性病感染风险的认知情况。

方法：2018 年在我国南京和苏州两市进行定性研究。研究人员对不同类型的跨性别女性进行关键知情者深入访谈(n = 14)和焦点小组访谈(n = 2)。访谈内容经转录和翻译后，用 Dedoose™ 定性资料软件进行编码、分析和解读。

结果：本研究发现中国跨性别女性与全球跨性别女性存在共性的经历是长期和极具挑战性的身份搜索，遭遇污名化和歧视性，难以获得跨性别人群特有的医疗服务以及无法满足心理健康需求等。但中国跨性别女性独有的特征包括自我性别认同中的术语使用，中国传统文化背景下对繁衍后代的期望以及将家庭和社会福祉置于个人成就之上的理想化状态。此外，该人群社交关系网稀疏分散和更加隐蔽。几乎所有受访者都经历过家庭排斥，对艾滋病

和性病感染的风险认知以及既往 HIV 检测率均很低。
结论：我国跨性别女性存在高度的社会排斥和歧视性，同时该人群无法获取特有的医疗服务。亟需扩大跨性别医疗服务的可及性和心理健康关怀，加强对艾滋病和性病的预防干预，最终改善我国跨性别女性人群的社会、医疗和心理健康问题。

Translated from English version into Chinese by Hong-Jing Yan

Identité, stigmatisation et risque du VIH chez les femmes transgenres : étude qualitative dans la province de Jiangsu en Chine

Zihan Yan, Jessica Lin, Wenjing Xiao, Keh-Ming Lin, Willi McFarland, Hongjing Yan, Erin Wilson

Résumé
Contexte: Les femmes transgenres présentent de multiples disparités à l'échelle mondiale, y compris le rejet social et la stigmatisation, l'infection du VIH et les problèmes de santé mentale non traités. Cependant, peu de données sur les femmes transgenres sont disponibles en Chine. Ainsi, cette étude vise à explorer les expériences des femmes transgenres sur l'identité sexuelle, la divulgation, la discrimination, les soins médicaux spécifiques aux transgenres et les perceptions du risque du VIH et d'infections sexuellement transmissibles (IST) en Chine.
Méthodes: En 2018, une étude qualitative a été menée dans les villes de Nanjing et Suzhou en Chine. Des entretiens avec des informateurs clés (*n* = 14) et des discussions de groupe (*n* = 2) avec diverses femmes transgenres ont été menés. Le texte a été transcrit et traduit, et l'équipe de recherche a utilisé le logiciel Dedoose™ pour le codage, l'analyse et l'interprétation.
Résultats: Les femmes transgenres chinoises partagent leurs expériences avec des femmes transgenres à travers le monde, ce qui inclut une recherche d'identité longue et difficile, la stigmatisation et la discrimination, un accès limité aux services spécifiques aux personnes transgenres et des besoins non satisfaits en soins de santé mentale. Les caractéristiques qui leur sont propres comprennent les termes utilisés pour l'auto-identification, les attentes culturelles en matière de reproduction et les idéaux plaçant le bien-être familial et sociétal au-dessus de l'épanouissement personnel. Les réseaux sociaux de cette population semblent clairsemés, dispersés et clandestins. Presque toutes les femmes interrogées ont été victimes de rejet familial. Les perceptions du risque de VIH et d'IST et les antécédents de dépistage du VIH étaient particulièrement faibles.
Conclusions: En Chine, les femmes transgenres sont confrontées à un rejet social élevé et à la discrimination, ainsi qu'à des besoins non satisfaits en matière de divers types de soins de santé. L'expansion des services spécifiques aux transgenres qui inclut les soins médicaux, les soins de santé mentale et la prévention du VIH et des IST, est justifiée pour améliorer la santé sociale, médicale et mentale des femmes transgenres en Chine.

Translated from English version into French by Paul Nyambo, revised by Thaïs Harvalias, through

TRANSLATORS
WITHOUT BORDERS

Гендерная идентичность, стигма и риск заражения ВИЧ среди трансгендерных женщин в провинции Цзянсу, Китай: качественное исследование

Цзыхань Янь, Джессика Линь, Вэньцзинь Сяо, Кэ-Мин Линь, Вилли Макфарланд, Хунцзин Янь, Эрин Уилсон

Резюме

Предпосылки: Трансгендерные женщины страдают от многих видов неравенства по всему миру, в том числе социального отторжения и стигмы, ВИЧ-инфекции и проблем с психическим здоровьем без соответствующего лечения. Однако, в Китае доступно достаточно мало данных о трансгендерных женщинах. Поэтому в данной работе были исследованы трансгендерные женщины в отношении их гендерной идентичности, раскрытия информации о сексуальной принадлежности, дискриминации, медицинской помощи характерной для трансгендерных женщин и восприятию риска ВИЧ и других инфекций, передаваемых половым путем (ИППП) в Китае.

Методы: в 2018 году в Нанкине и городе Сучжоу, Китай, было проведено количественное исследование. Были проведены интервью с ключевыми информаторами ($n = 14$) и обсуждения в фокусных группах ($n = 2$) с различными трансгендерными женщинами. Текст был транскибирован и переведен, и исследовательская группа использовала программное обеспечение *Dedoose*™ для кодирования, анализа и интерпретации.

Результаты: китайские трансгендерные женщины подвергаются всему тому же, что и транссексуальные женщины во всем мире, включая длительный и сложный поиск идентичности, стигму и дискриминацию, плохой доступ к трансгендер-специфическим услугам и неудовлетворенные потребности в психиатрической помощи. Уникальными для них являются термины, используемые для самоидентификации, культурно-обусловленные ожидания в отношении деторождения и идеалы доминирования интересов семейного и общественного блага над личной реализацией. Социальные сети этого населения предсталяются редкими, рассеянными и подпольными. Семейное неприятие испытали почти все респонденты. Осознание риска заражения ВИЧ и ИППП и история тестирования на ВИЧ были заметно снижеными.

Выводы: трансгендерные женщины в Китае сталкиваются с серьезным социальным неприятием и дискриминацией, а также неудовлетворенной потребностью в различных видах здравоохранения. Для решения проблем социального, медицинского и психического здоровья трансгендерных женщин в Китае необходимо расширение услуг, ориентированных на транссексуалов, включая оказание медицинской помощи с учетом гендерных факторов, охрану психического здоровья и профилактику ВИЧ/ИППП.

Translated from English version into Russian by Daniel Casado Rodriguez, revised by Ana Valera, through

TRANSLATORS
WITHOUT BORDERS

Identidad, estigma y riesgo de VIH entre mujeres transgénero: un estudio cualitativo en la provincia de Jiangsu, China

Zihan Yan, Jessica Lin, Wenjing Xiao, Keh-Ming Lin, Willi McFarland, Hongjing Yan, Erin Wilson

Resumen

Introducción: las mujeres transgénero sufren múltiples disparidades en todo el mundo, entre ellas el rechazo social, estigma, infecciones por VIH y problemas de salud mental no tratados. Sin embargo, hay pocos datos disponibles sobre mujeres transgénero en China. Por lo tanto, el objetivo de este estudio fue analizar las experiencias en identidad de género de las mujeres transgénero, la divulgación, la discriminación, la atención médica específica para personas transgénero y las percepciones del riesgo de VIH e infecciones de transmisión sexual (ITS) en China.

Metodología: se realizó un estudio cualitativo en las ciudades de Nanjing y Suzhou, China, en 2018. Se llevaron a cabo entrevistas con informantes clave (n = 14) y discusiones de grupos de debate (n = 2) con diversas mujeres transgénero. El texto fue transcrito y traducido, y el equipo de investigación utilizó el software *Dedoose*™ para la codificación, el análisis y la interpretación.

Resultados: Las mujeres transgénero chinas comparten experiencias con mujeres transgénero de todo el mundo, incluyendo una larga y desafiante búsqueda de identidad, estigma y discriminación, poco acceso a servicios específicos para personas transgénero y necesidades de atención de salud mental no cubiertas. Entre sus características exclusivas se incluyen los términos utilizados para la autoidentificación, las expectativas de reproducción forjadas culturalmente y los ideales de colocar el bienestar familiar y social por encima de la realización personal. Las redes sociales de esta parte de la población parecen dispersas y clandestinas. Casi todas las encuestadas experimentaron rechazo familiar. La percepción del riesgo de VIH e ITS y el historial de pruebas de VIH fueron notablemente bajos.

Conclusiones: Las mujeres transgénero en China se enfrentan a un alto nivel de rechazo social y de discriminación, junto con una necesidad de varios tipos de atención médica que no se ve cubierta. La ampliación de los servicios específicos para personas transgénero, incluida la atención médica que afirme el género, la atención de la salud mental y la prevención del VIH y las infecciones de transmisión sexual, está justificada para abordar la salud social, médica y mental de las mujeres transgénero en China.

Translated from English version into Spanish by Maria Petrenko, revised by Alexander Somin, through

TRANSLATORS
WITHOUT BORDERS
